# Numerical investigation of the cavitation bubble near the solid wall with a gas-entrapping hole based on a fully compressible three-phase model

**DOI:** 10.1016/j.ultsonch.2023.106531

**Published:** 2023-07-23

**Authors:** Jianyong Yin, Yongxue Zhang, Xueyu Qi, Lei Tian, Dehong Gong, Mingkai Ma

**Affiliations:** a Electrical Engineering College, Guizhou University, Guiyang 550025, China; b College of Mechanical and Transportation Engineering, China University of Petroleum-Beijing, Beijing 102249, China; c Beijing Key Laboratory of Process Fluid Filtration and Separation, China University of Petroleum-Beijing, Beijing 102249, China

**Keywords:** Cavitation bubble, Solid wall with a gas-entrapping hole, Compressible three-phase model, The liquid jet, OpenFOAM

## Abstract

The solid surface with several cavities containing gas strongly influences the bubble’s dynamical behaviors. To reveal the underlying physical mechanism of the cavitation bubble near a rigid boundary with a gas-entrapping hole, a fully compressible three-phase model, accounting for the three-phase volume transport equation, was implemented in OpenFOAM. The predicted bubble shape was validated with the corresponding experimental photos, and good agreement was achieved. The bubble’s primary physical features (e.g., the expanding shock wave, upward and downward liquid jet, and high-pressure region) are well reproduced, which helps understand the underlying mechanisms. The numerical results show that the solid wall with a gas-entrapping hole could affect the morphology of both the bubble and liquid jet, as well as shortens the bubble's first oscillation period in comparison to an intact rigid wall. The relationship among the prolongation factor, the standoff distance, and the relative size ratio is analyzed. It is found the prolongation factor increases as the relative size ratio decrease. As the standoff distance decreases, the gas entrapping hole plays a significant role in the oscillation period of the bubble. The current model can be further extended to reveal the microscopic mechanism of aeration avoiding cavitation damage and investigate the interaction between air bubbles and cavitation bubbles, which is of great interest to practical applications.

## Highlights

• A fully compressible three-phase model accounting for the three-phase volume transport equation is implemented.
• The solid wall with a gas-entrapping hole could affect the morphology of both the bubble and liquid jet, as well as changes their flow direction in comparison to an intact rigid wall.
• The effects of the stand-off distance *γ* and relative sizes *ε* on the minimum bubble radius, bubble first collapse time, and maximum temperature are discussed and summarized.

## 1 Introduction

Cavitation, generated by the rupture of the liquid with a dramatic local energy release or pressure drop, is commonly accompanied by noise, vibration, erosion, and so on, leading to the degradation of the performance of the hydraulic equipment [1], [2], [3], [4], [5]. By contrast, it also presents promise in many industrial fields, such as seabed exploration in ocean engineering [6], [7], periodontal pocket surface cleaning [8], drug and gene delivery [9], [10], urinary stone ablation [11], inactivating viruses [12], as well as water treatment [13].

There have been extensive investigations on the cavitation bubble dynamics near boundaries of various shapes in recent decades [14], [15], [16], [17], [18], [19]. The liquid jet and shock wave from the cavitation bubble collapsing are considered responsible for the damage to the structure. When the cavitation bubble occurs in the vicinity of a solid object, both the bubble and liquid jet move toward the boundary, as well as its dynamic behaviors depend on the standoff distance *γ*. With the auxiliary of a high-speed camera operating at 1,000,000 frames/s, Philipp and Lauterborn [20] reported that the formation of the liquid jet and the emission of shock waves under different *γ*, and found solid surface damage could be observed for *γ* less than 2.0. Han et al. [21] investigated the coalescence of multiple cavitation bubbles (two or three bubbles) in the vicinity of a solid wall, even in an extreme situation where the bubble is tight to the solid boundary. They found the liquid jet produced by the multiple bubbles collapsing also flowed toward the rigid wall. Brujan et al. [22] reported the behavior of the bubble at the corner of two perpendicular solid walls and found the bubble migrated in the direction of the jet. Li et al. [23] studied the collapsing dynamics of a bubble near two-connected walls with an obtuse angle. Comparing the two perpendicular walls, they found the jet deviation from the horizontal direction was only in a much shorter range. In addition, when the bubble is adjacent to the free surface rather than the solid boundary, both the bubble and the jet will be repelled away from the free surface due to an opposite-direction pressure gradient [24], [25], [26], [27]. Li et al. [27] investigated the bubble-bursting behaviors near a free surface and showed the liquid jet formation at different dimensionless distances. Zhang et al. [28] reported the bubble features between the free surface and solid wall and discovered that the jet velocity was smaller than that in the vicinity of a solid-only wall. Similarly, Huang et al. [25] analyzed the characteristics of bubble collapse and rebound under different conditions of free surface and rigid wall. According to the morphology of the bubble, three patterns were identified, i.e., single annular bubble without spike, single annular bubble with spike, and double annular bubble with spike.

The above literature reviews reveal the dynamical behaviors of the cavitation bubble near an intact boundary (solid wall or free surface). Meanwhile, a few scholars have expanded the research fields to study the features of the bubble near a discontinuous boundary. He et al. [29] numerically investigated the effect of the stand-off distance on the bubble near a breach. The results revealed that the stand-off distances defined the inrush patterns, including inrush with a bursting bubble, inrush with a gourd-shaped bubble, and inrush with a floating bubble. Liu et al. [30] numerically and experimentally investigated the jet of the cavitation bubble under the combined effect of a free surface and a fixed flat plate with a circular opening. Furthermore, Chen et al. [31] showed the bubble dynamics near a double-layer plate with a circular hole and divided the bubble morphology into five categories according to the feature of the inrushing bubble. Trummler et al. [32] revealed the effect of the crevice geometry on jet formation, wave dynamics, and wall-pressure distribution.

Recently, Gonzales-Avila et al. [33] released that the air trapped inside cavities could repel the bubbles away from the boundary surface, therefore preventing cavitation damage. Similarly, Sun et al. [34] also found that the solid surface with several cavities containing gas enabled the direction of motion of the jet produced by the bubble collapsing to flow away from the wall. Subsequently, their newest work [35] found that the solid wall with a gas entrapping hole also changed the migration direction of the cavitation bubble. As mentioned in the above literature, compared with the intact solid wall, the rigid surface with a small hole containing gas can affect the direction of the movement of the bubble and change the trajectory of the jet. But, the physical mechanism of the jet direction conversion and the bubble dynamical behaviors near the solid wall with a gas-entrapping hole are not fully revealed. Due to the experimental difficulties in guaranteeing that the centroids of the bubble and gas hole are on the same level, especially for the smaller gas hole diameter. Hence, the effect of the gas hole diameters on the bubble dynamics has not been well studied yet.

The dynamical behaviors of the cavitation bubble near the solid boundary with a gas entrapping hole bring enormous challenges to theoretical and numerical research. The earliest theoretical research on bubble dynamics could date back to the twentieth century. Until now, the classical Rayleigh-Plesset (RP) equation [36] can be used to well describe the collapse of a spherical bubble, which is generated in an infinite fluid (term “free field” bubble). However, it will be unsuitable for describing the bubble near various boundaries due to the incompressible assumption, resulting in the neglect of the energy lost by bubble collapse-induced pressure waves [37]. Extended from the RP equation, the Keller-Miksis equation further accounted for the compressibility of the fluid outside the bubble to achieve more accurate results [38]. In addition, Prosperetti and Lezzi [39], [40] also developed a model with consideration of the compressibility of the fluid based on the perturbation theory. Geers and Hunter [41] developed a doubly asymptotic approximation model specialized for the single underwater bubble in a free field with consideration of fluid compressibility. Although these above models could play an important role in the theoretical study of bubble dynamics in free fields, they are still invalid when investigating the bubble near boundaries [42]. Besides, the recent theoretical research from Zhang et al. [42] demonstrates that their model can simultaneously account for the effects of various boundaries, inter-bubble interactions, fluid compressibility, viscosity, and surface tension, which can accurately predict the key bubble dynamics features and shows potential in exploring more sophisticated physics and mechanisms behind bubble dynamics. However, the complete boundaries are used in their theoretical models, i.e., free surfaces or solid wall. The effect of the combination of the solid wall and free surface on bubble dynamics is deprived.

Most of the current numerical research on the cavitation bubbles near various boundaries is based on the compressible two-phase (gas–liquid, or vapor–liquid) model [26], [43], [44], [45], [46], [47]. In our previous study [48], a compressible two-phase solver has been implemented to investigate the thermodynamic properties of the bubble pairs. To reveal the physical mechanism of the cavitation bubble near a rigid boundary with a gas entrapping hole, the compressible two-phase model is further expanded to incorporate the three-phase (gas, vapor, and liquid) volume transport equation (more information can be seen in Appendix A). One objective of the present study is to develop a fully compressible three-phase solver based on the OpenFOAM-5.0 platform [49]. Another objective is concerned with the dynamical behaviors of the cavitation bubble on the nearby solid wall with a gas entrapping hole, which may be an important application to reduce cavitation damage by controlling the direction of the jet. The physical model and numerically implemented process are presented in Section 2. Validation of the compressible three-phase model by comparing the current results with the experimental phenomena is shown in Section 3. In Section 4, the dynamics of the cavitation bubble near a wall with a gas entrapping hole at different stand-off distances are analyzed and discussed. Afterward, the effect of the gas hole diameter on the bubble is investigated. Finally, the conclusions are provided in Section 5.

## 2 Physical model

### 2.1 Governing equations

Based on the free, open-source CFD software OpenFOAM-5.0, a compressible three-phase (i.e., gas, vapor, and liquid) model was established. A set of non-linear governing equations were solved within the entire computational domain and the specific implementation process was presented in the following.

The mass equations for each phase can be formulated as:

(1) $$\frac{\partial \left(\alpha_{i}\rho_{i}\right)}{\partial t}+\nabla \cdot \left(\alpha_{i}\rho_{i}U\right)=0\left(i=g,v,l\right)$$

where $\rho_{i}$ and $\alpha_{i}$ are the density and volume fraction of each phase. the subscript *i* represents the gas phase (*g*), vapor phase (*v*), and liquid phase (*l*), respectively. $\alpha_{i}$ satisfies the conservation law *α_l_* + *α_v_* + *α_g_* = 1.0 and 0 < *α_l_*, *α_v_*, *α_g_* less than 1. These phase interfaces, i.e., the gas–liquid interface, the vapor–liquid interface, and the gas–vapor interface, are captured by the volume of fluid (VOF) method, which has superior mass conservation characteristics and has been widely applied in literature [48], [50], [51], [52], [53]. In the VOF method, only one transport equation derived from Eq (1) for the liquid phase needs to be solved for the two-phase flow. However, three transport equations have to be solved in turn for the more complex three-phase flow and are defined below (the relative information can be referred to in Appendix A):

(2) $$\begin{array}{c} \frac{\partial \alpha_{l}}{\partial t}+\nabla \cdot \left(\alpha_{l}U\right)+\nabla \cdot \left(\alpha_{l}\alpha_{v}\left(U_{l}-U_{v}\right)+\alpha_{l}\alpha_{g}\left(U_{l}-U_{g}\right)\right)=-\frac{\alpha_{l}}{\rho_{l}}\left[\frac{D\rho_{l}}{\mathrm{Dt}}\right]\left(1-\alpha_{l}\right)\\ +\alpha_{l}\left[\frac{\alpha_{v}}{\rho_{v}}\frac{D\rho_{v}}{\mathrm{Dt}}+\frac{\alpha_{g}}{\rho_{g}}\frac{D\rho_{g}}{\mathrm{Dt}}\right]+\alpha_{l}\left(\nabla \cdot U\right)\left(\text{liquid phase}\right) \end{array}$$

(3) $$\begin{array}{c} \frac{\partial \alpha_{v}}{\partial t}+\nabla \cdot \left(\alpha_{v}U\right)+\nabla \cdot \left(\alpha_{v}\alpha_{l}\left(U_{v}-U_{l}\right)+\alpha_{v}\alpha_{g}\left(U_{v}-U_{g}\right)\right)=-\frac{\alpha_{v}}{\rho_{v}}\left[\frac{D\rho_{v}}{\mathrm{Dt}}\right]\left(1-\alpha_{v}\right)\\ +\alpha_{v}\left[\frac{\alpha_{l}}{\rho_{l}}\frac{D\rho_{l}}{\mathrm{Dt}}+\frac{\alpha_{g}}{\rho_{g}}\frac{D\rho_{g}}{\mathrm{Dt}}\right]+\alpha_{v}\left(\nabla \cdot U\right)\left(\text{vapor phase}\right) \end{array}$$

(4) $$\begin{array}{c} \frac{\partial \alpha_{g}}{\partial t}+\nabla \cdot \left(\alpha_{g}U\right)+\nabla \cdot \left(\alpha_{g}\alpha_{l}\left(U_{g}-U_{l}\right)+\alpha_{g}\alpha_{v}\left(U_{g}-U_{v}\right)\right)=-\frac{\alpha_{g}}{\rho_{i}}\left[\frac{D\rho_{g}}{\mathrm{Dt}}\right]\left(1-\alpha_{g}\right)\\ +\alpha_{g}\left[\frac{\alpha_{l}}{\rho_{l}}\frac{D\rho_{l}}{\mathrm{Dt}}+\frac{\alpha_{v}}{\rho_{v}}\frac{D\rho_{v}}{\mathrm{Dt}}\right]+\alpha_{g}\left(\nabla \cdot U\right)\left(\text{gas phase}\right) \end{array}$$

where these additional artificial compression terms,$\nabla \cdot \left(\alpha_{l}\alpha_{v}\left(U_{l}-U_{v}\right)+\alpha_{l}\alpha_{g}\left(U_{l}-U_{g}\right)\right)$, $\nabla \cdot \left(\alpha_{v}\alpha_{l}\left(U_{v}-U_{l}\right)+\alpha_{v}\alpha_{g}\left(U_{v}-U_{g}\right)\right)$,$\nabla \cdot \left(\alpha_{g}\alpha_{l}\left(U_{g}-U_{l}\right)+\alpha_{g}\alpha_{v}\left(U_{g}-U_{v}\right)\right)$ introduced by Weller [54] guarantee the sharpness of the phase interface, which is only active in the interface range due to the presence of *α_l_α_v_*, *α_l_α_g_*, and *α_g_α_v_*. (***U****_l_* - ***U****_v_*), (***U****_l_* – ***U****_g_*), and (***U****_v_* – ***U****_g_*) are the artificial compression velocity between any two phases, acting normally to the interface [55].

(5) $$U_{\mathrm{rij}}=U_{i}-U_{j}=\min\left[C_{a}\left|U\right|,\max\left(\left|U\right|\right)\right]\frac{\nabla \alpha_{i}}{\left|\nabla \alpha_{i}\right|}\left(i,j=l,v,g\right)$$

where *C*_a_ is the regulating factor of phase interface compressibility. *C*_a_ = 1 is adopted in the current simulation, which ensures conservative compression.

The momentum equation is as follows:

(6) $$\frac{\partial \left(\rho U\right)}{\partial t}+\nabla \cdot \left(\rho \mathrm{UU}\right)=-\nabla p_{-rgh}-gh\nabla \rho +\nabla \cdot \tau +F_{s}$$

where *ρ* and ***g*** are the mixture density and the gravitational acceleration, respectively. $p_{-rgh}=p-\rho gh$ is the pressure excluding the hydrostatic pressure; ***F****_s_* is the surface tension force between phase pairs (i.e., gas–vapor, gas–liquid, and vapor–liquid); *τ* is the viscous stress tensor and written as:

(7) $$\tau =\mu \nabla U+\mu {\left(\nabla U\right)}^{T}-\frac{2}{3}\nabla \cdot \mathrm{UI}$$

where *μ* and ***I*** are the mixture of dynamic viscosity and the unit tensor, respectively.

The mixture density *ρ* and dynamic viscosity *μ* are obtained as follows:

(8) $$\rho =\alpha_{l}\rho_{l}+\alpha_{v}\rho_{v}+\alpha_{g}\rho_{g}$$

(9) $$\mu =\alpha_{l}\mu_{l}+\alpha_{v}\mu_{v}+\alpha_{g}\mu_{g}$$

The total energy equation expressed in terms of temperature *T* for three phases [56] reads:

(10) $$\begin{array}{c} \frac{\partial \left(\rho T\right)}{\partial t}+\nabla \cdot \left(\rho TU\right)+\left(\frac{\alpha_{l}}{C_{\mathrm{pl}}}+\frac{\alpha_{v}}{C_{\mathrm{pv}}}+\frac{\alpha_{g}}{C_{\mathrm{pg}}}\right)\left[\frac{\partial \left(\rho K\right)}{\partial t}+\nabla \cdot \left(\rho TU\right)+\nabla \cdot \left(Up\right)\right]\\ -\left(\frac{\alpha_{l}\lambda_{L}}{C_{\mathrm{pl}}}+\frac{\alpha_{v}\lambda_{v}}{C_{\mathrm{pv}}}+\frac{\alpha_{g}\lambda_{g}}{C_{\mathrm{pg}}}\right)\left(\nabla^{2}T\right)=0 \end{array}$$

where $C_{\mathrm{pi}}\left(i=l,g,v\right)$ and $\lambda_{i}\left(i=l,g,v\right)$ are the heat capacity and thermal conductivity of three-phase, respectively. *K* is the kinetic energy with $K=U^{2}/2$.

Different forms of the equation of state are adopted for closing the above governing equations and calculating phase densities, which are based on pressure and temperature variations. For gas and vapor two phases, the ideal gas equation of state is employed as follows:

(11) $$\rho_{i}=\frac{p}{R_{i}T}\left(i=v,g\right)$$

(12) $$\psi_{i}=\frac{d\rho_{i}}{\mathrm{dp}}=\frac{1}{R_{i}T}$$

where the gas constant$R_{g}$ = 284.75 J/(kg·K) and$R_{v}$ = 461.6 J/(kg·K). $\psi_{i}$ is the compressibility of both gas and vapor phases.

For the liquid phase, the equation of state for the pure liquid introduced by Shin et al. [57] is applied:

(13) $$\rho_{l}=\frac{p+p_{c}}{K_{c}\left(T+T_{c}\right)}$$

(14) $$\psi_{l}=\frac{d\rho_{l}}{\mathrm{dp}}=\frac{1}{K_{c}\left(T+T_{c}\right)}$$

where $\psi_{l}$ is the compressibility of the liquid phase. More information on the details of the liquid phase equation of state can be seen in our previous work [48].

**Fig. 1:**
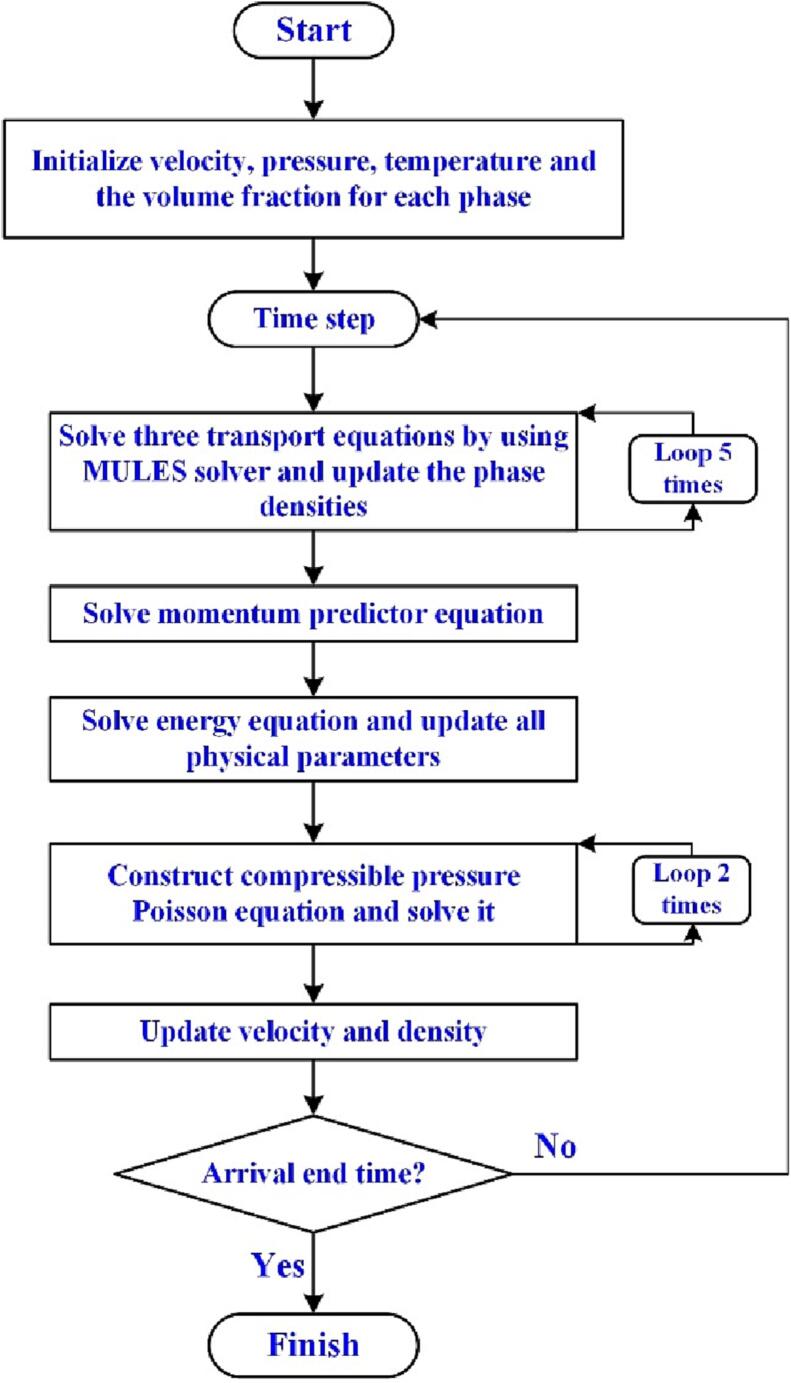
Flow chart of three-phase compressible solver.

The solution process of the three-phase compressible solver is shown in Fig. 1. In the VOF method, an explicit MULES solver [58], combining the artificial compression term and flux corrected transport (FCT) [59] technology, is used to solve three transport equations (Eqs. (2)-(4)). The coupled pressure–velocity variables are solved using the PISO algorithm [60], which is widely applied to the solution of transient problems. The first-order implicit Euler scheme and second-order Guass TVD scheme with van Leer limiter are employed for the time term and convection term, respectively. An adjustable time step is applied for improving the calculation speed. The maximum Courant number (maxCo) is less than 0.4 to avoid the numerical dissipation for compressible cavitation flow. To reduce the iteration error, the tolerances for pressure, velocity, temperature, and volume fraction residual convergence criterion under each iteration are set small enough to 10^-14^.

### 2.2 Numerical setup

**Fig. 2:**
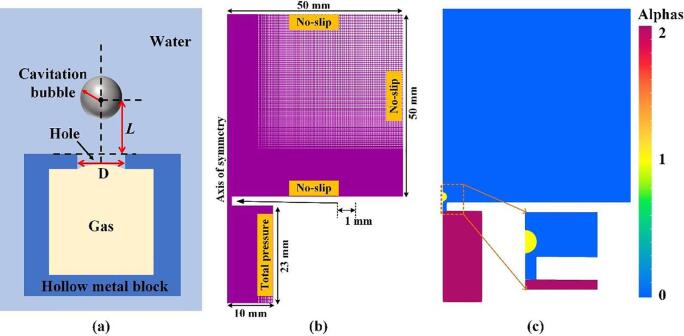
(a) The experimental schematic; (b) the axisymmetric calculation region, boundary conditions, and mesh distribution; (c) three-phase volume fraction visualization.

**Fig. 3:**
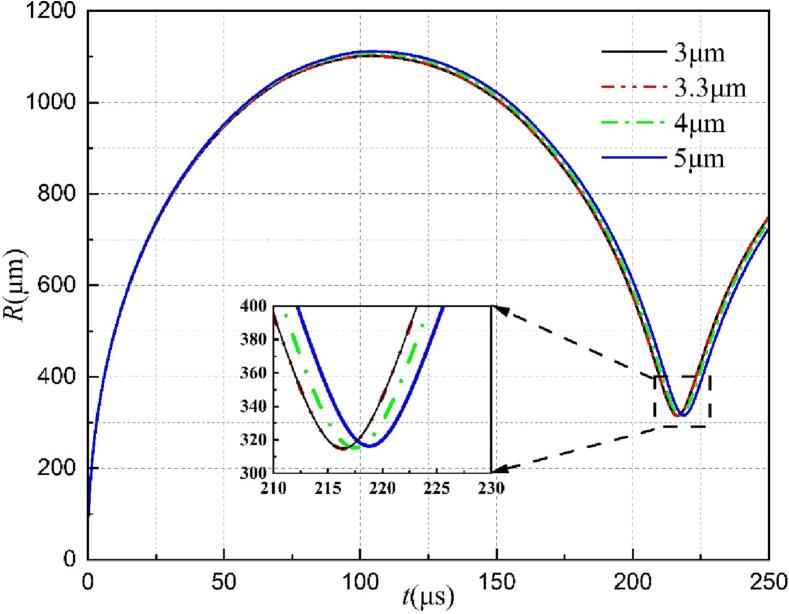
The variation of the cavitation bubble radius over time with four different grids.

The experimental results of a cavitation bubble in the vicinity of the rigid surface with a gas entrapping hole from Sun et al. [35] are selected as the reference for constructing the corresponding calculated domain. The related experimental schematic and several key bubble parameters are shown in Fig. 2(a). As seen, a hollow metal block with a hole containing gas was placed in a water tank and the cavitation bubble was generated by using the laser generator. To reduce interference, the centroids of the hollow metal block, the hole, and the cavitation bubble were ensured to be approximately on the same horizontal line. *D* is the hole diameter and *L* is the distance from the cavitation bubble center to the hole. More information about the experimental setup can be found in Ref [35]. In this study, a 2D axisymmetric calculation domain is adopted for reducing the computational cost. The calculation region, boundary conditions, and mesh distribution are shown in Fig. 2(b). The dimensions of both the computational zone and the hole are determined based on the initial experimental conditions in Ref [35]. The uniform and dense grids were generated near the cavitation bubble (0–2 mm and 0–3 mm in the *×* and *y* direction, respectively), where the large shape deformation occurs. Also, relatively sparse and widely spaced grids were created where is away from the bubble for improving computation efficiency. To check the grid independency, the interactions of the cavitation bubble and the gas entrapping hole are simulated using four different grids with the smallest cell size of 3 μm (total number of grids 1510965), 3.3 μm (total number of grids 1310145), 4 μm (total number of grids 1008165), and 5 μm (total number of grids 745915). The mesh information and simulation results under different grids are presented in Table 1. As seen, the maximum deviation of both the *R*_max_ and *R*_min_ is less than 0.1% when the total number of grids is equal to 1310145. Furthermore, the variations of bubble radius over time with four different meshes are given in Fig. 3. When the smallest grid is less than 3.3 μm, the predicted bubble radius and collapse time are almost the same. Hence, the mesh with the cell number 1,310,145 (3.3 μm) is adopted in this paper to perform the following simulations. Additionally, the physical properties of the gas, the vapor (i.e., the cavitation bubble), and the surrounding liquid are provided in Table 2.

**Table 1:** The analysis of grid independence.

Grid options	Total number	Smallest cell size (μm)	*R*_max_(μm)	Deviation for *R*_max_(%)	*R*_min_(μm)	Deviation for *R*min(%)
Fine grid	1,510,965	3	1101.72	–	314.89	–
Intermidiate grid	1,310,145	3.3	1100.92	0.07	314.94	0.02
Slightly coarse grid	1,008,165	4	1105.93	0.46	315.43	0.16
Coarse grid	745,915	5	1111.71	0.52	316.31	0.28

**Table 2:** The physical properties of the three phases.

Parameter	Gas phase	Vapor phase	Liquid phase
*ρ*(kg/m^3^)	1	0.0171	998.2
*μ*(Pa·s)	1.589 × 10^-5^	9.75 × 10^-6^	9.982 × 10^-4^
*C*_p_(J/kg·K)	1000	1862.6	4180
*λ*(W/m·K)	0.026	0.02	0.677
*σ*(N/m)	0.07		

As mentioned previously (Eqs. (2)-(4)), the sum of the volume fraction ($\alpha_{i}$) of the three phases is equal to 1. However, three-volume fraction fields are very difficult to visualize in one image. Thus, the Alphas, being an indicator of different phases, is introduced to visualize all phases, as shown in Fig. 2(c). Here, Alphas = 0 represents the liquid phase (blue); Alphas = 1 indicates the vapor phase (yellow) and 2 is the air phase (red). The strength of the interaction of the cavitation bubble and the gas hole is closely related to the relative distance (*L*) and hole diameter size (D). Thus, the dimensionless parameters are defined as follows:

The standoff distance *γ*

(15) $$\gamma =\frac{L}{R_{\max}}$$

The relative size ratio *ε*

(16) $$\varepsilon =\frac{D/2}{R_{\max}}$$

where *R*_max_ is the maximum radii of the cavitation bubble.

## 3 Three-phase model validation

### 3.1 Validation via the experiment of the cavitation bubble near a rigid boundary with a gas-entrapping hole

To validate the developed three-phase compressible model, the comparison was performed first using the experimental results of the cavitation bubble near the rigid wall with a gas-entrapping hole [35]. The experimental observations ((al)-(a10)) and the current simulation results ((b1)-(b10)) were compared frame by frame to reveal the interaction of the cavitation bubble and the gas entrapping hole, as shown in Fig. 5. In addition, the corresponding pressure and velocity fields are presented in Fig. 7. In the current simulation, the initial parameters (*D* and *L*) are same as the experimental data, i.e., D = 2 mm and *L* = 1.22 mm. The initial radii, pressure, and temperature within the simulated bubble are assumed to be 0.088 mm, 6 × 10^7^ Pa, and 593.15 K, respectively. Besides, the ambient pressure and temperature for the liquid phase are set to be 101,325 Pa and 293.15 K, respectively.

**Fig. 4:**
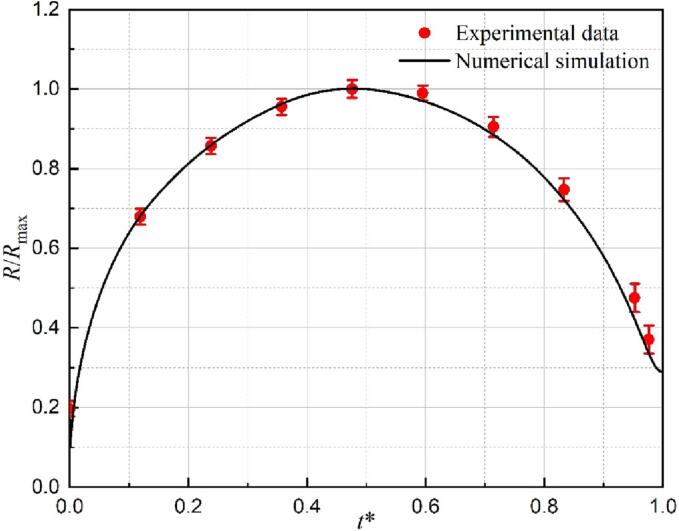
Comparison of the predicted bubble radius with the experimental data in Ref [35] at γ = 1.22.

**Fig. 5:**
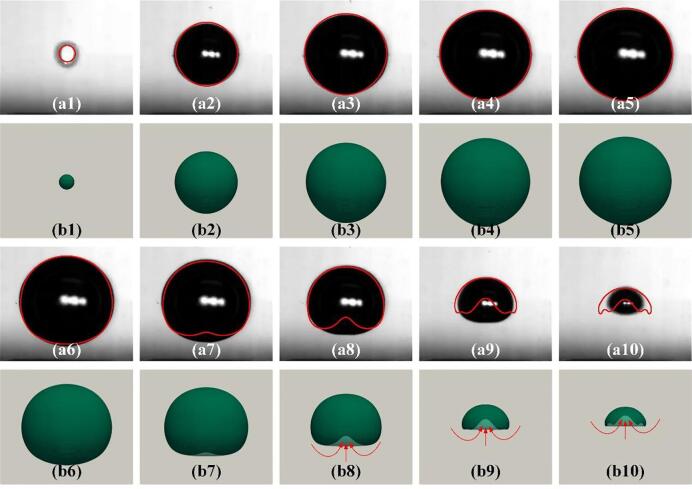
Comparison of the predicted bubble shape with the experimental photos. (a1) - (a10) the experimental results in Ref. [35] with γ = 1.22; (b1) - (b10) the current simulation results with γ = 1.22. a(b)1 t*=0; a(b)2 t*=0.119; a(b)3 t*=0.238; a(b)4 t*=0.357; a(b)5 t*=0.476; a(b)6 t*=0.595; a(b)7 t*=0.714; a(b)8 t*=0.833; a(b)9 t*=0.952; a(b)10 t*=0.976.

The dimensionless method introduced by Han et al. [61] is employed, i.e., R* (*R*/*R*_max_) and t*(*t*/*T*_fc1_), here *T*_fc1_ is the time of the cavitation bubble first collapse. A quantitative comparison between the experimental data [35] (red dot) and the current simulation (black line) is shown in Fig. 4. As seen, the predicted results are in good agreement with the experimental data. Particularly, the predicted bubble radius during collapse is slightly smaller than that in the experiment due to the appearance of a bubble non-spherical shape, leading to increased errors in experimental measurements. Meanwhile, the maximum error of the numerical bubble radius is only 6.27%.

Furthermore, the computational results are superimposed on the experimental images as red circles, as shown in Fig. 5. The cavitation bubble grows with a spherical shape (frames (a1-a2)) during initial expansion since the bubble is far from the gas hole (γ = 1.22). Subsequently, the bottom of the cavitation bubble could be observed as significantly elongated during expansion, as shown in frames (a3-a5). During the collapse, the cavitation bubble bottom shrinks faster and moves away from the hole (frames (a7-a10)). Simultaneously, an upward jet appears in the simulation, as shown in frames (b8-b10). The predicted bubble size is slightly different from the experimental observations mainly because only the 2D simulation results are presented for the sake of observing the internal bubble shape change. Nevertheless, the interactions between the cavitation bubble and the rigid wall with a gas entrapping hole are well reproduced by the current numerical simulation. Overall, the simulated results agree well with the experimental phenomena.

### 3.2 Validation via the experiment of the cavitation bubble near the complete rigid wall

**Fig. 6:**
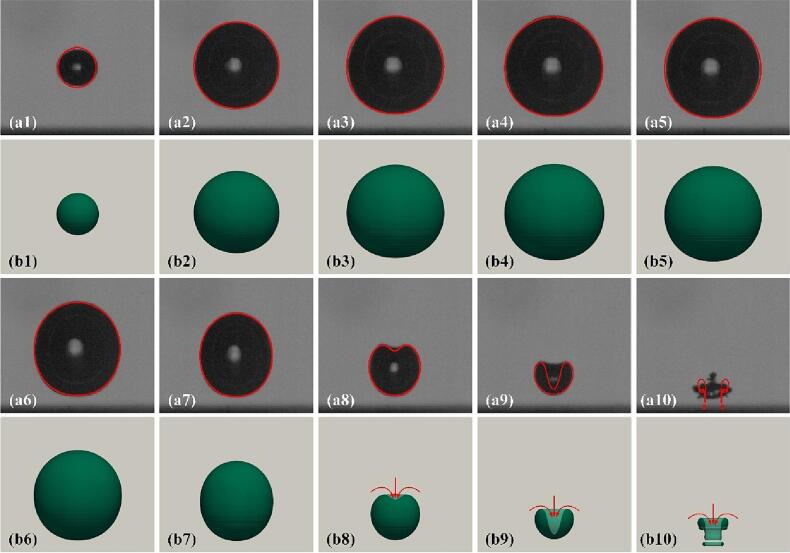
Comparison of the predicted bubble shape with the experimental photos. (a1) - (a10) the experimental results from our previous works with *γ* = 1.22. (b1) - (b10) the current simulation results with *γ* = 1.22. a(b)1 t*=0.037; a(b)2 t*=0.222; a(b)3 t*=0.370; a(b)4 t*=0.481; a(b)5 t*=0.593; a(b)6 t*=0.704; a(b)7 t*=0.815; a(b)8 t*=0.926; a(b)9 t*=0.963; a(b)10 t*=1.0.

In this section, the experiments of the single cavitation bubble near the complete rigid wall from our previous work [46] are selected as further reference for model validation. The initial parameters inside and outside the bubble are identical to those in Section 3.1. The simulated results are compared with our previous experiments with *γ* = 1.22, as shown in Fig. 6. The presence of the solid wall induces an asymmetric collapse of the cavitation bubble, resulting in a large pressure difference between the top and bottom of the bubble. Thus, a liquid jet formed and moved towards the rigid wall (see frames b8-b10), which is different from those shown in Fig. 5 (appearance of upward jet). The cavitation bubble near the complete solid wall has been widely reported [43], [46], [51], [62], [63]. Thus, the cavitation bubble dynamic behaviors in the case of the solid boundary with a gas entrapping hole will be investigated in focus in the next Section. Overall speaking, as can be seen in Fig. 6, the predicted bubble shapes are in good agreement with the experimental results.

## 4 Result and discussion

### 4.1 The dynamics of the cavitation bubble near a wall with a gas entrapping hole with *γ* = 1.22

**Fig. 7:**
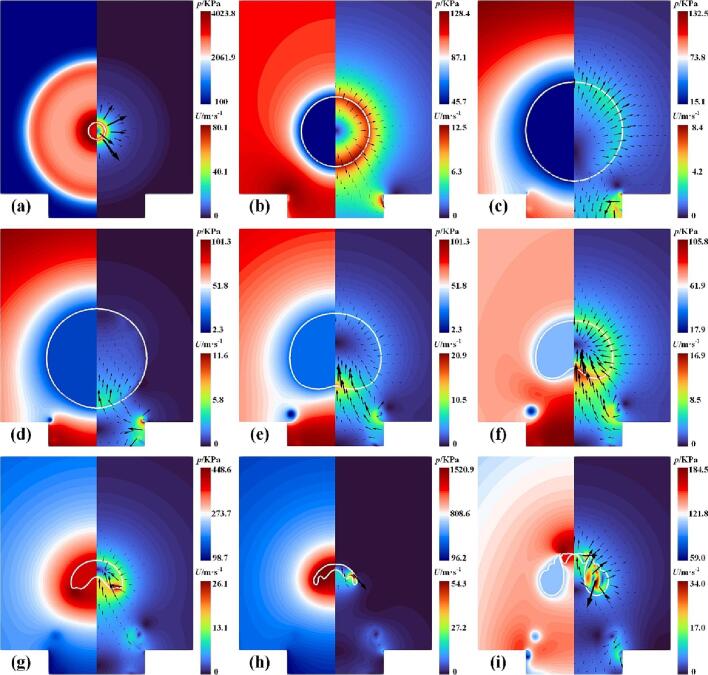
Snapshots of the pressure and velocity fields at (a) *t* = 1 μs, (b) *t* = 24 μs, (c)*t* = 72 μs, (d) *t* = 96 μs, (e) *t* = 143 μs, (f) *t* = 167 μs, (g) *t* = 191 μs, (h) *t* = 200 μs, and (i) *t* = 220 μs with *γ* = 1.22.

**Fig. 8:**
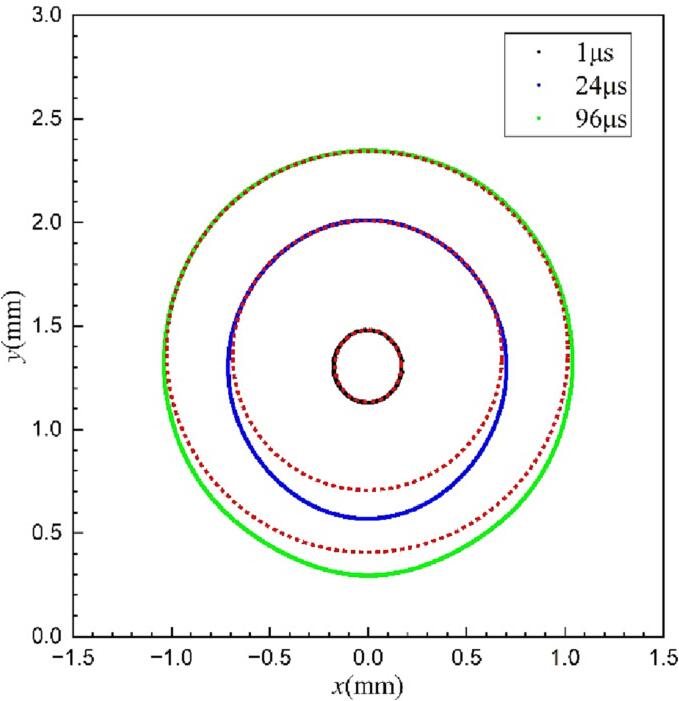
Bubble contours at different times during expansion with *γ* = 1.22.

The dynamics of a cavitation bubble near the rigid wall with a gas entrapping hole at *γ* = 1.22 are investigated and discussed first. Fig. 7 shows the contours of the pressure and velocity with *γ* = 1.22, where the solid white circle indicates the vapor–liquid interface. As seen in Fig. 7(a), the cavitation bubble expands outward due to initially high internal pressure and emits the expanding shock wave. Due to the relatively small distance (*γ* = 1.22), the sides of the lower half of the bubble are subjected to a strong obstruction of the hole wall surface, causing the bottom of the bubble to be elongated, as shown in Fig. 8. These red dashed standard circles are used as a reference for the evolution of the bubble shape at different times. Also, the upper part of the bubble is almost unaffected by the wall and expands outwards in a circular shape, as shown in Fig. 7(b) and (c). Fig. 7(d) presents that the bubble expands to the maximum volume, where the lower bubble surface has contracted because of the high-pressure region between the bubble bottom and the gas entrapping hole. During collapse, an upward jet forms and the liquid enters the interior of the bubble, as seen in Fig. 7(e) and (f). Fig. 7(g) shows the high-pressure zone emerges around the collapsing bubble and then the bubble reaches its minimum radius, where the upward jet has not penetrated the bubble, as shown in Fig. 7(h). Fig. 7(i) illustrates the jet has pierced through the rebound bubble and a new high-pressure area appears above the bubble.

**Fig. 9:**
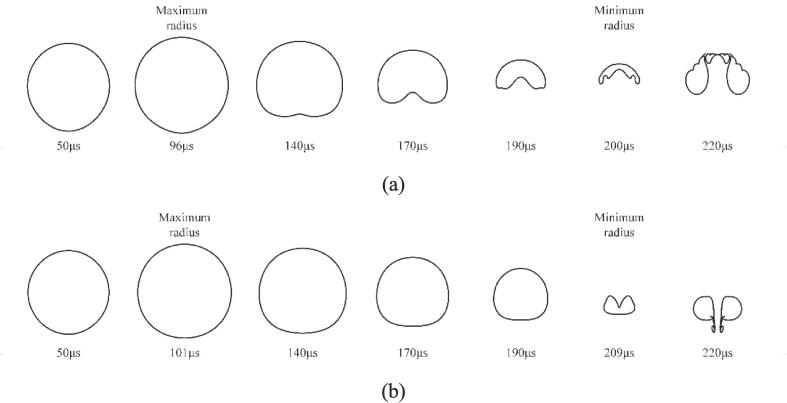
Evolution of the bubble shape for the bubble near the solid wall with a hole at *γ* = 1.22, (a) the hole filled with the gas, (b) the hole filled with the liquid.

**Fig. 10:**
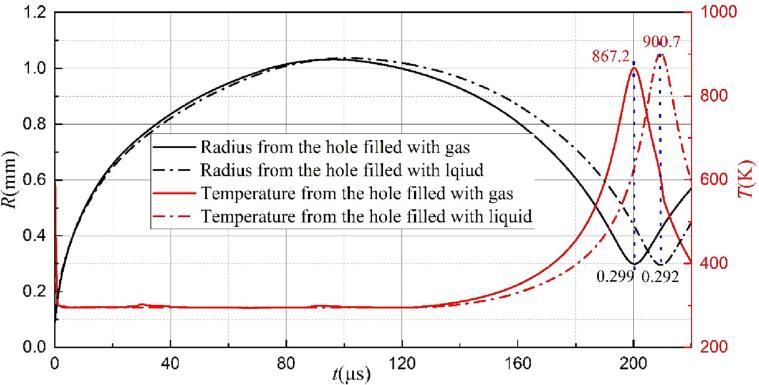
Bubble radius (black line) and the maximum temperature (red line) obtained from the case of the hole filled with the gas (solid line) and the liquid (dot line).

Fig. 9 compares the bubble shapes simulated near a rigid wall with (a) and without (b) the gas, i.e., the hollow metal blocks (Fig. 2(a)) are filled with gas and liquid, respectively. In the case of the solid wall with a hole containing gas, an upward jet formed and flowed far from the hole surface, as shown in Fig. 9(a). In contrast, Fig. 9(b) shows the movement of the jet towards the boundary in the case of the hole containing liquid. In other words, the solid wall with a gas-entrapping hole could affect the morphology of the liquid jet and changes its direction. Additionally, the bubble collapse time from the initial to the first minimal radius with the presence of the gas is slightly shorter than that without gas, since the bubble in the case of the gas hole can collapse to a relatively larger radius, as shown in Fig. 10. Brujan et al. [64] also found that the cavitation bubble lifetime decreases with the increase of the minimum radius. As investigated in our previous work [48], a smaller minimum radius corresponds to a higher maximum temperature. In Fig. 10, the maximum temperature from the case of the hole containing liquid is higher.

### 4.2 The dynamics of the cavitation bubble near a wall with a gas entrapping hole with *γ* = 1.91

**Fig. 11:**
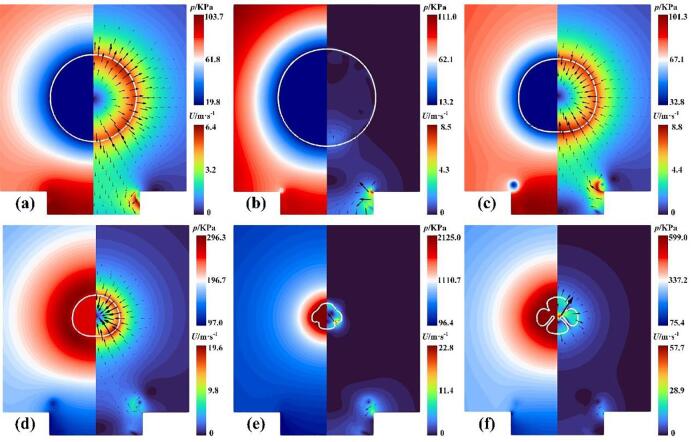
Snapshots of the pressure and velocity fields at (a) *t* = 50 μs, (b) *t* = 99 μs, (c)*t* = 161 μs, (d) *t* = 191 μs, (e) *t* = 203 μs, and (f) *t* = 210 μs with *γ* = 1.91.

When *γ* increases to 1.91, the strength of the interaction between the cavitation bubble and the gas entrapping hole is relatively weakened. The corresponding contours of the pressure and velocity are shown in Fig. 11. The cavitation bubble expands outward (Fig. 11(a)) and eventually reaches its maximum volume (Fig. 11(b)). At this moment, the lower part of the bubble did not appear to shrink, which is different from the simulation situations shown in Fig. 7(d). During the collapse of the bubble, the upward liquid jet did not occur, where the rate of collapse on the upper surface of the bubble is almost identical to that of the lower surface, as shown in Fig. 11(c) and (d). A similar experimental phenomenon without the upward jet has been observed by Sun et al. [35]. Fig. 11(e) presents the bubble collapses to its minimum size. Subsequently, the bubble re-expands in all directions due to the absence of the liquid jet, as shown in Fig. 11(f).

**Fig. 12:**
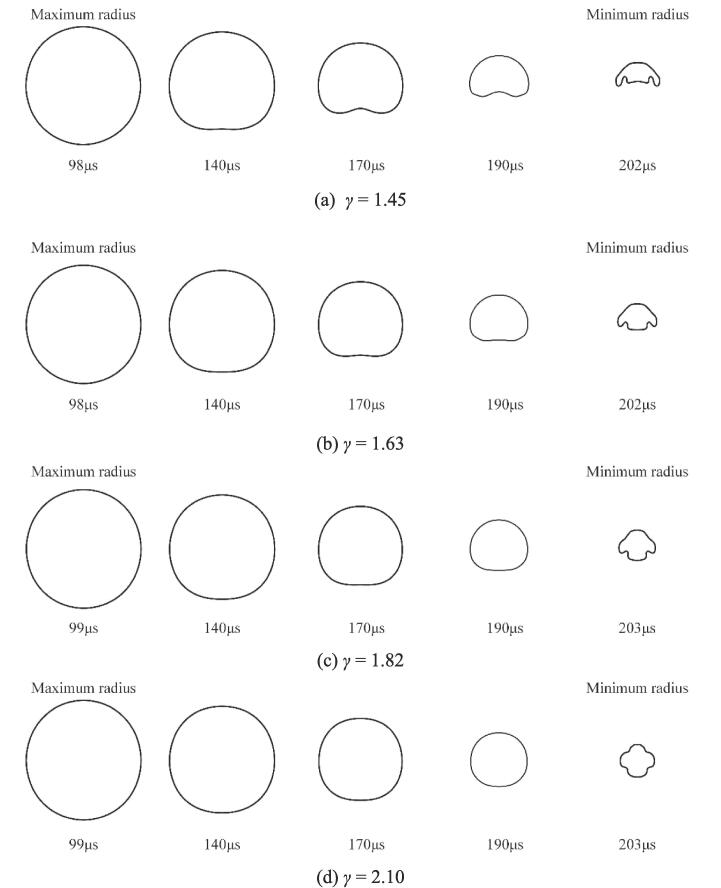
Evolution of the bubble shape under different *γ*.

**Fig. 13:**
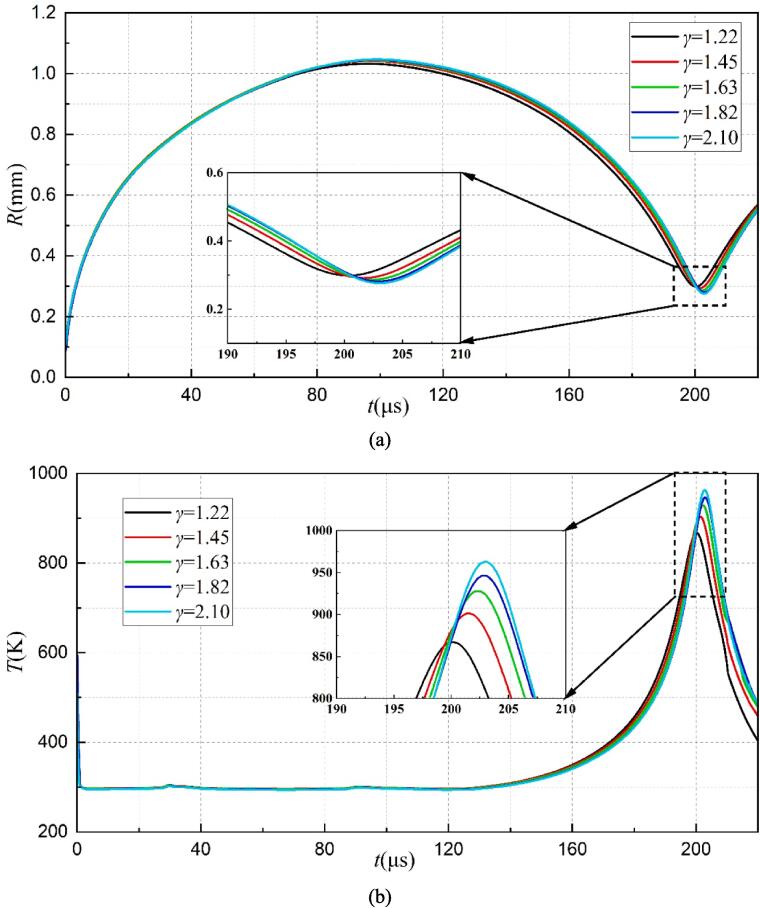
The variation of the bubble radius (a) and the maximum temperature (b) inside the bubble versus time under different *γ*.

The evolution of the bubble shape under four different *γ* is shown in Fig. 12. As seen, the upward jet still appears during the bubble collapse at *γ* = 1.45. In addition, there is no upward jet for *γ ≥* 1.63. Fig. 13 shows the variation of bubble radius and the maximum temperature within the bubble over time for various *γ*. With the increase of *γ*, the bubble's first collapse time increases but the minimum radius decreases, as shown in Fig. 13(a). Moreover, the maximum temperature inside the bubble also increases as *γ* increases as shown in Fig. 13(b), since the weakened obstructive effect of the rigid wall with the gas-entrapping hole and more energy being focused in the bubble, which is noticeable by the smaller minimum radius.

**Fig. 14:**
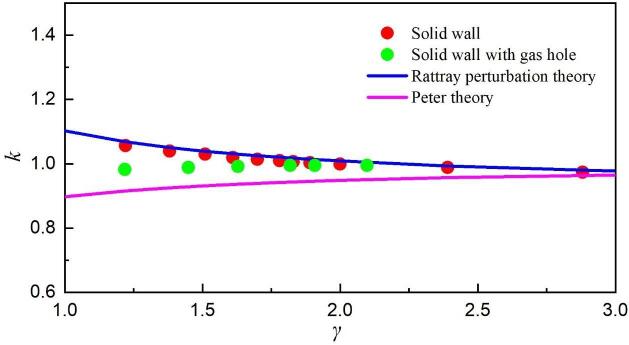
The relationship between the bubble’s first collapse time and *γ*. Red dot is the bubble near the solid wall and green dot is the bubble near the solid wall with a gas-entrapping hole.

Numerous studies have shown that the intact rigid wall could prolong the bubble's first collapse time while the free surface will shorten the oscillation period of the bubble [35]. The prolongation factor k_p_ from Rattray’s perturbation theory [65] is defined as the ratio of the bubble collapse time near the solid wall to Rayleigh’s collapse time, which can be simply expressed by Eq. (17). Similarly, a shortened factor k_s_ derived by Peter at al. [66] is defined as the ratio of the bubble collapse time near the free surface to the Rayleigh collapse time, which can be described by Eq. (18). To better elucidate the potential relationship between the bubble's first collapse time and *γ*, the relationship between them under the two wall conditions, i.e., an intact solid wall and the solid boundary with a gas entrapping hole, is shown in Fig. 14. Here, k is defined as the ratio of the bubble first collapse time to the Rayleigh’s collapse time.

(17) $$k_{p}=0.915\left(1+\frac{0.205}{\gamma }\right)$$

(18) $$k_{s}=1-\frac{0.102}{\gamma }$$

As seen, the simulation results in the case of the solid wall with a gas-entrapping hole are in the middle of the two theoretical results, meaning that the bubble is affected by both the solid wall and the gas hole. Besides, as *γ* decreases, the gas hole plays an increasingly significant role in the oscillation period of the bubble since the results are more in favor of the Peter theory, which is also supported by the presence of upward jets.

### 4.3 The effect of the relative size ratio (*ε*)

**Fig. 15:**
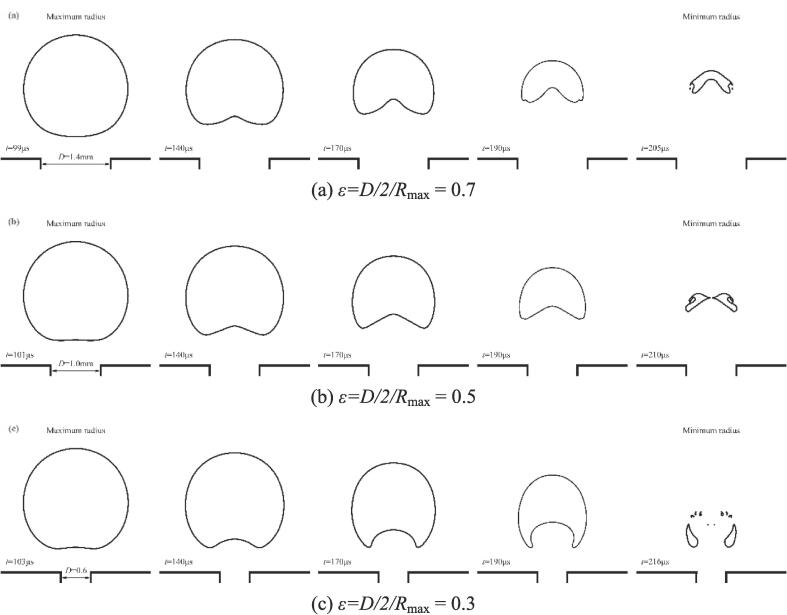
Comparison of the bubble shape under different size ratios (*ε*) with *γ* = 1.22.

**Fig. 16:**
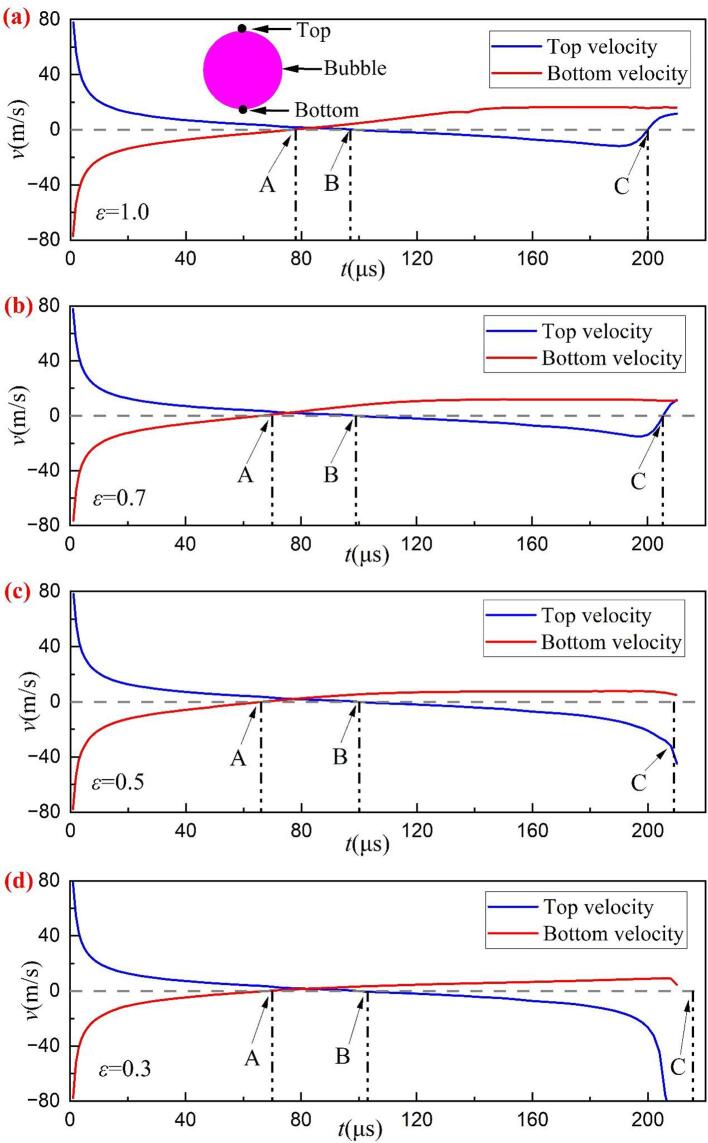
Time histories of the velocity at the top and bottom of the bubble under different *ε*.

In this section, the dynamical behaviors of the cavitation bubble at different relative size ratios *ε* are shown in Fig. 15. Four different *ε* are chosen and the bubble shape for *ε* = 1 is shown in Fig. 9(a). Moreover, the time histories of the velocity at the top and bottom of the bubble under different *ε* are provided in Fig. 16. Here, velocity upward is specified as the positive direction. Points A, B, and C are the times of zero velocity at the bubble bottom, zero velocity at the bubble top, and the bubble minimum radius, respectively. The top and bottom locations are also shown in Fig. 16(a) (see the pink bubble). As shown in Fig. 15, the upward jets appear for all hole diameters during bubble collapse. For the cases of *ε* = 1 (Fig. 9(a)) and 0.7 (Fig. 15(a)), the upward jets do not pierce the bubble when it collapses to the minimum radius. At this moment, the top velocity is zero while the bottom speed is not zero, as shown in point C in Fig. 16(a) and (b). At the initial stage of bubble re-expansion, both top and bottom velocities are greater than zero, meaning that the rebound bubble is still moving away from the gas hole.

**Fig. 17:**
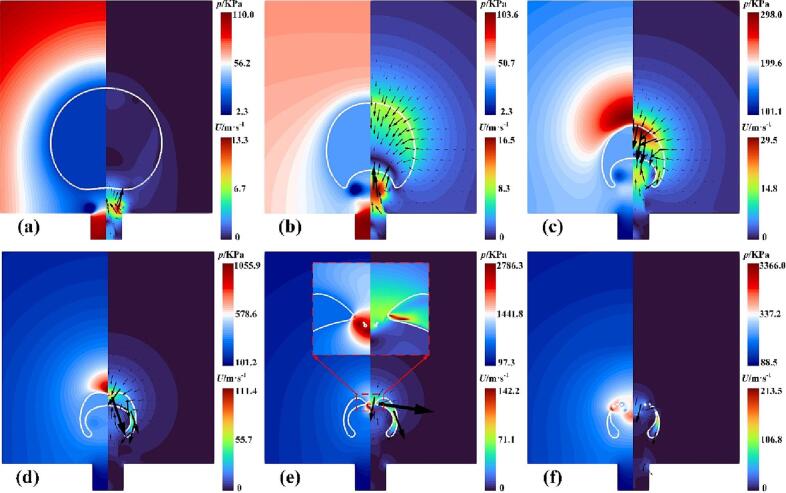
Snapshots of the pressure and velocity fields at (a) *t* = 103 μs, (b) *t* = 170 μs, (c)*t* = 200 μs, (d) *t* = 206 μs, (e) *t* = 210 μs, and (f) *t* = 215 μs with *γ* = 1.22 and *ε* = 0.3.

For *ε* = 0.5 (Fig. 15(b)) and 0.3 (Fig. 15(c)), the liquid jets penetrated the bubble when the minimum radius was reached. Meanwhile, the top velocity is much less than 0, indicating that the top of the bubble may move toward the hole. To better understand the final stage of the bubble collapse for the case of *ε* = 0.3, the pressure and velocity fields during collapse are shown in Fig. 17. Fig. 17(a) shows the bubble reaches its maximum volume. At this moment, the velocity of the bubble top is almost zero (shown in point B in Fig. 16(d)) while the bottom becomes flat due to shrinkage. Fig. 17(b) shows the upward liquid jet forms due to the high pressure in the gas hole. In the late stage of bubble collapse, the other high-pressure region occurs near the top of the bubble (Fig. 17(c)), driving the liquid jet down into the bubble (Fig. 17(d)). Subsequently, the two liquid jets with opposite directions penetrates the bubble and turns the bubble into a toroidal shape, as shown in Fig. 17(e). When the upward jet from the high pressure in the gas hole encounters the downward jet produced by the high pressure near the bubble top, the gas within the bubble between the two jets is squeezed out and flows into the remaining side of the bubble, forming the gas jet as shown in the partial enlargement in Fig. 17(e). Lechner et al. [67] pointed out that the gas jet was related to the shock front whose velocity exceeded the local sound speed. Compared to the state with a large *ε* (Fig. 7), the bubble collapse appears as two liquid jets with opposite directions (Fig. 17(e)), implying that the effect of the rigid wall can not be neglected for a small *ε*. Finally, the toroidal bubble continues collapsing to the minimum radius, as shown in Fig. 17(f).

**Fig. 18:**
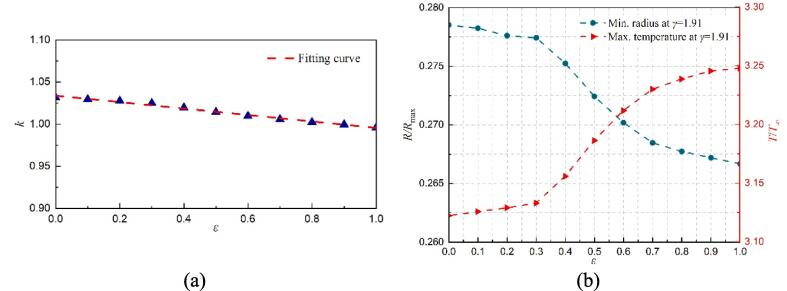
The effect of *ε* on the bubble's first collapse time, minimum radius, and maximum temperature with *γ* = 1.91. (a) the relationship of the collapse time and *ε*, (b) minimum radius and maximum temperature within the cavitation bubble.

The influence of *ε* on the prolongation factor (*k*), minimum radius, and maximum temperature for the first collapse at *γ* = 1.91 are shown in Fig. 18. As can be seen in Fig. 18(a), the prolongation factor (*k*) increases as *ε* decreases. And their values are all smaller than that from the special case where the solid surface without the gas entrapping hole (*ε* = 0). Thus, compared with the intact solid surface, the rigid wall with a gas-entrapping hole can shorten the bubble's first oscillation period. In addition, the minimum radius also increases as *ε* decreases, as shown in Fig. 18(b). As previously discussed, a smaller minimum radius corresponds to a higher maximum temperature. Therefore, the maximum temperature increases as *ε* increases.

**Fig. 19:**
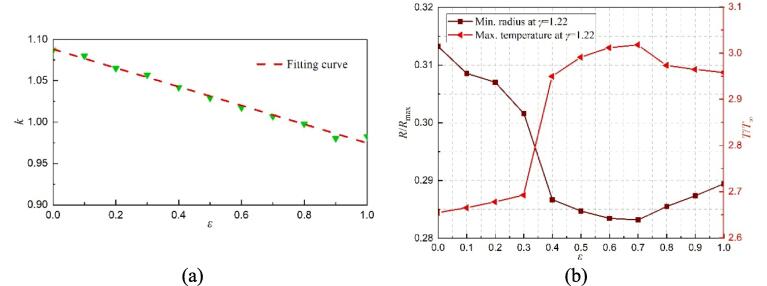
The effect of *ε* on the bubble's first collapse time, minimum radius, and maximum temperature with *γ* = 1.22. (a) the relationship of the collapse time and *ε*, (b) minimum radius and maximum temperature within the cavitation bubble.

For a smaller *γ* = 1.22, the effect of *ε* on various parameters is shown in Fig. 19. Similar to Fig. 18(a), the prolongation factor (*k*) also increases as *ε* decreases. However, the minimum radius decreases first and then increases with the increase of *ε*. For *ε* ≤ 0.5, as shown in Fig. 17, two liquid jets with opposite directions occur during the bubble's first collapse, indicating that the bubble is affected by the combination of the solid wall and the gas entrapping hole. As *ε* decreases, the more prominent the obstruction of the solid wall, eventually corresponding to a larger minimum radius.

For *ε* > 0.5, as shown in Fig. 7, only an upward liquid jet appears, demonstrating the gas-entrapping hole plays a more significant role. Besides, as *ε* increases, the minimum radius also increases, but the rate of increase is much smaller than that from the case of the combined effect of the solid wall and the gas hole. Finally, the maximum temperature increases first and then decreases with the increase of *ε*.

In addition, the current compressible three-phase model can be further extended to reveal the microscopic mechanism of aeration avoiding cavitation damage [68] and investigate the interaction between air bubbles and cavitation bubbles [69], which is of great interest to practical applications.

## 5 Conclusion

In this study, a fully compressible three-phase model was implemented to investigate the dynamical feature of the cavitation bubble near the rigid boundary with a gas-entrapping hole. Validation was conducted by comparing the predicted bubble shape with the related experimental data. The bubble’s primary physical features during the first cycle are well reproduced based on the current numerical model. Moreover, the effects of stand-off distance *γ* and relative size *ε* on the bubble's dynamical behaviors are analyzed and discussed. The main conclusions can be drawn as follows:

1. In comparison to an intact rigid wall, the solid wall with a gas entrapping hole could affect the morphology of both the bubble and liquid jet, as well as changes their flow direction.
2. As *γ* decreases, the gas entrapping hole plays an increasingly significant role in the oscillation period of the bubble. There is no significant upward jet appearing during the bubble collapse when *γ* ≥ 1.63.
3. The prolongation factor (*k*) increases as *ε* decreases. Compared with the intact solid surface, the rigid wall with a gas-entrapping hole can shorten the bubble's first oscillation period. For *ε* ≤ 0.5, two liquid jets with opposite directions occur during collapse, indicating that the bubble is affected by the combination of the solid wall and the gas-entrapping hole. For *ε* > 0.5, only an upward liquid jet appears, demonstrating the gas hole plays a more significant role.

## Declaration of Competing Interest

The authors declare that they have no known competing financial interests or personal relationships that could have appeared to influence the work reported in this paper.

## Appendix A

The mass equations for the liquid, vapor, and gas phases, respectively can be expressed as follows:

(A-1) $$\frac{\partial \alpha_{l}}{\partial t}+\nabla \cdot \left(\alpha_{l}U\right)=-\frac{\alpha_{l}}{\rho_{l}}\frac{d\rho_{l}}{\mathrm{dt}}$$

(A-2) $$\frac{\partial \alpha_{v}}{\partial t}+\nabla \cdot \left(\alpha_{v}U\right)=-\frac{\alpha_{v}}{\rho_{v}}\frac{d\rho_{v}}{\mathrm{dt}}$$

(A-3) $$\frac{\partial \alpha_{g}}{\partial t}+\nabla \cdot \left(\alpha_{g}U\right)=-\frac{\alpha_{g}}{\rho_{g}}\frac{d\rho_{g}}{\mathrm{dt}}$$

Merging the above Eqs (A-1), (A-2), and (A-3):

(A-4) $$\nabla \cdot U=-\left(\frac{\alpha_{l}}{\rho_{l}}\frac{d\rho_{l}}{\mathrm{dt}}+\frac{\alpha_{v}}{\rho_{v}}\frac{d\rho_{v}}{\mathrm{dt}}+\frac{\alpha_{g}}{\rho_{g}}\frac{d\rho_{g}}{\mathrm{dt}}\right)$$

Expanding the convection term of Eq. (A-4):

(A-5) $$\frac{\partial \alpha_{l}}{\partial t}+\alpha_{l}\nabla \cdot U+U\cdot \nabla \alpha_{l}=-\frac{\alpha_{l}}{\rho_{l}}\frac{d\rho_{l}}{\mathrm{dt}}$$

Merging the Eqs (A-4) and (A-5):

(A-6) $$\frac{\partial \alpha_{l}}{\partial t}+U\cdot \nabla \alpha_{l}=-\frac{\alpha_{l}}{\rho_{l}}\frac{d\rho_{l}}{\mathrm{dt}}\left(1-\alpha_{l}\right)+\alpha_{l}\left(\frac{\alpha_{v}}{\rho_{v}}\frac{d\rho_{v}}{\mathrm{dt}}+\frac{\alpha_{g}}{\rho_{g}}\frac{d\rho_{g}}{\mathrm{dt}}\right)$$

Adding $\alpha_{l}\nabla \cdot U$ into Eq. (A-6), the final transport equation for the liquid phase can be written as:

(A-7) $$\frac{\partial \alpha_{l}}{\partial t}+\nabla \cdot \left(\alpha_{l}U\right)=-\frac{\alpha_{l}}{\rho_{l}}\frac{d\rho_{l}}{\mathrm{dt}}\left(1-\alpha_{l}\right)+\alpha_{l}\left(\frac{\alpha_{v}}{\rho_{v}}\frac{d\rho_{v}}{\mathrm{dt}}+\frac{\alpha_{g}}{\rho_{g}}\frac{d\rho_{g}}{\mathrm{dt}}\right)+\alpha_{l}\nabla \cdot U$$

Similarly, the final transport equations for both the vapor phase and gas phase can be obtained:

(A-8) $$\frac{\partial \alpha_{v}}{\partial t}+\nabla \cdot \left(\alpha_{v}U\right)=-\frac{\alpha_{v}}{\rho_{v}}\frac{d\rho_{v}}{\mathrm{dt}}\left(1-\alpha_{v}\right)+\alpha_{v}\left(\frac{\alpha_{l}}{\rho_{l}}\frac{d\rho_{l}}{\mathrm{dt}}+\frac{\alpha_{g}}{\rho_{g}}\frac{d\rho_{g}}{\mathrm{dt}}\right)+\alpha_{v}\nabla \cdot U$$

(A-9) $$\frac{\partial \alpha_{g}}{\partial t}+\nabla \cdot \left(\alpha_{g}U\right)=-\frac{\alpha_{g}}{\rho_{g}}\frac{d\rho_{g}}{\mathrm{dt}}\left(1-\alpha_{g}\right)+\alpha_{g}\left(\frac{\alpha_{v}}{\rho_{v}}\frac{d\rho_{v}}{\mathrm{dt}}+\frac{\alpha_{l}}{\rho_{l}}\frac{d\rho_{l}}{\mathrm{dt}}\right)+\alpha_{g}\nabla \cdot U$$

Adding the additional artificial term proposed by Weller [54], which guarantees the sharpness of the phase interface. Finally, the transport equations for the three phases implemented in OpenFOAM are as:

(A-10) $$\begin{array}{c} \frac{\partial \alpha_{l}}{\partial t}+\nabla \cdot \left(\alpha_{l}U\right)+\nabla \cdot \left[\alpha_{l}\alpha_{v}\left(U_{l}-U_{v}\right)+\alpha_{l}\alpha_{g}\left(U_{l}-U_{g}\right)\right]=-\frac{\alpha_{l}}{\rho_{l}}\frac{d\rho_{l}}{\mathrm{dt}}\left(1-\alpha_{l}\right)\\ +\alpha_{l}\left(\frac{\alpha_{v}}{\rho_{v}}\frac{d\rho_{v}}{\mathrm{dt}}+\frac{\alpha_{g}}{\rho_{g}}\frac{d\rho_{g}}{\mathrm{dt}}\right)+\alpha_{l}\nabla \cdot U \end{array}$$

(A-11) $$\begin{array}{c} \frac{\partial \alpha_{v}}{\partial t}+\nabla \cdot \left(\alpha_{v}U\right)+\nabla \cdot \left[\alpha_{v}\alpha_{l}\left(U_{v}-U_{l}\right)+\alpha_{v}\alpha_{g}\left(U_{v}-U_{g}\right)\right]=-\frac{\alpha_{v}}{\rho_{v}}\frac{d\rho_{v}}{\mathrm{dt}}\left(1-\alpha_{v}\right)\\ +\alpha_{v}\left(\frac{\alpha_{l}}{\rho_{l}}\frac{d\rho_{l}}{\mathrm{dt}}+\frac{\alpha_{g}}{\rho_{g}}\frac{d\rho_{g}}{\mathrm{dt}}\right)+\alpha_{v}\nabla \cdot U \end{array}$$

(A-12) $$\begin{array}{c} \frac{\partial \alpha_{g}}{\partial t}+\nabla \cdot \left(\alpha_{g}U\right)+\nabla \cdot \left[\alpha_{g}\alpha_{l}\left(U_{g}-U_{l}\right)+\alpha_{g}\alpha_{l}\left(U_{g}-U_{g}\right)\right]=-\frac{\alpha_{g}}{\rho_{g}}\frac{d\rho_{g}}{\mathrm{dt}}\left(1-\alpha_{g}\right)\\ +\alpha_{g}\left(\frac{\alpha_{v}}{\rho_{v}}\frac{d\rho_{v}}{\mathrm{dt}}+\frac{\alpha_{l}}{\rho_{l}}\frac{d\rho_{l}}{\mathrm{dt}}\right)+\alpha_{g}\nabla \cdot U \end{array}$$
